# CAMbase – A XML-based bibliographical database on Complementary and Alternative Medicine (CAM)

**DOI:** 10.1186/1742-5581-4-2

**Published:** 2007-04-03

**Authors:** Thomas Ostermann, Hartmut Zillmann, Christa K Raak, Arndt Buessing, Peter F Matthiessen

**Affiliations:** 1 Department of Medical Theory and Complementary Medicine, University of Witten/Herdecke, Gerhard-Kienle-Weg 4, 58313 Herdecke, Germany; 2 Intelligent Data Management, 49084 Osnabrück, Germany

## Abstract

The term "Complementary and Alternative Medicine (CAM)" covers a variety of approaches to medical theory and practice, which are not commonly accepted by representatives of conventional medicine. In the past two decades, these approaches have been studied in various areas of medicine. Although there appears to be a growing number of scientific publications on CAM, the complete spectrum of complementary therapies still requires more information about published evidence. A majority of these research publications are still not listed in electronic bibliographical databases such as MEDLINE. However, with a growing demand by patients for such therapies, physicians increasingly need an overview of scientific publications on CAM. Bearing this in mind, CAMbase, a bibliographical database on CAM was launched in order to close this gap. It can be accessed online free of charge or additional costs.

The user can peruse more than 80,000 records from over 30 journals and periodicals on CAM, which are stored in CAMbase. A special search engine performing syntactical and semantical analysis of textual phrases allows the user quickly to find relevant bibliographical information on CAM. Between August 2003 and July 2006, 43,299 search queries, an average of 38 search queries per day, were registered focussing on CAM topics such as acupuncture, cancer or general safety aspects. Analysis of the requests led to the conclusion that CAMbase is not only used by scientists and researchers but also by physicians and patients who want to find out more about CAM.

Closely related to this effort is our aim to establish a modern library center on Complementary Medicine which offers the complete spectrum of a modern digital library including a document delivery-service for physicians, therapists, scientists and researchers.

## Background

The term 'Complementary and Alternative Medicine (CAM)' covers a variety of approaches to medical theory and practice such as homeopathy, herbal medicine, naturopathy, anthroposophical medicine, to mention but a few, which are commonly not accepted by representatives of conventional medicine [1]. In contrast to this definition, several investigations have shown a rise in the use of CAM in almost all western industrial countries in the last two decades [2,3]. Many complementary therapies have therefore been the subject of medical research. One very important step in the research on CAM in Germany were the government funded projects "'Unconventional Methods in the Fight against Cancer"' (German abbreviation: UMK) and " 'Unconventional Medical Approaches"' (German abbreviation: UMR) from 1986–1996 [4,5]. In total, 31 projects were funded by these initiatives which eventually resulted in the establishment of a number of small research groups. These concentrated on special areas of CAM and managed to build up an academic research network [6]. However, they soon realized that the majority of CAM research publications were (and still are) difficult to find in electronic databases like MEDLINE. This is due to two major factors:

1. **CAM-Literature is widespread in various sources: **Because of a long tradition of complementary therapies in Germany, especially in the fields of homeopathy and naturopathy going back as far as the 18th century, a variety of journals developed into communication tools over the course of time. In Germany today, we have more than a hundred journals on CAM. However, only a few of these journals are listed in MEDLINE, which covers about 4,500 international journals. This is a language problem on the one hand; on the other hand it is also affected by the heterogeneous nature of the articles published in some of these journals. To make things even worse, CAM research findings have been communicated in monographs, proceedings and books, the so called 'grey literature', which is not listed in any established electronic database for medicine. This problem was confirmed early on by an investigation [7], which discovered that searches of MEDLINE for CAM generally resulted in between 17% (Homeopathy) and 51% (Acupuncture) of total papers published.

2. **A widely accepted Thesaurus for CAM in its entirety does not exist **regarding its heterogenity, CAM has not developed a sufficient culture of a controlled vocabulary to classify CAM-literature [8]. Although some promising efforts have been made [9], this is still an unsolved problem. In addition, the conventional MESH-Keywords of MEDLINE do not adequately map the contents of CAM-Literature, which means that even though there is bibliographical data on CAM in electronic databases, a researcher might use the wrong keywords in his search strategy resulting in his not finding the required data.

Aware of this situation, some research groups built up their own local bibliographical database concentrating on their specific needs and research topics such as Homeopathy (Munich and Essen), Anthroposophical Medicine (Witten/Herdecke), Spa Science (Bad Elster), Traditional Chinese Medicine (KIKOM) and Music Therapy (Witten/Herdecke) using locally available technical software such as LIDOS, REFMAN, ENDNOTE or MS-ACCESS. With an increasing number of researchers in the field of CAM, the demand emerged for a database which integrates these various literature sources on CAM. As a result, the CAMbase-project [10] was initiated by the Chair of Medical Theory and Complementary Medicine at Witten/Herdecke University and an open source online-database was created [11].

After a short description of the technical background of CAMbase, this article describes the usage-profile of CAMbase with regards to the access of CAMbase, search strategies used, search topics of CAMbase users and various future aspects of CAM-related digital resources are discussed.

## Realization of the CAMbase-Project

The initial situation implied a list of requirements, which were considered when the CAMbase project started. These were:

• Already existing electronic resources for CAM-Literature, which currently are only accessible offline (e.g. research group databases), should be integrated easily without much technical effort with regard to the structure of the bibliographical data.

• The search options should be adaptable both for experienced users, who want to perform a special search strategy, e.g. for systematic reviews, and also for those, who simply want to find out more about a topic, which at that moment might not be paraphrased very precisely.

• The mining and processing of the data should allow an electronic data exchange with other databases in standardized protocols with the use of common in – and output – styles.

• The search screen should be integrated without presupposition into existing http/web environments.

With regards to problems of infrastructure of peer to-peer networks and in accordance with [12], we decided to import the partners' data in a structured set-up into a central database with strategically separated sub-sets [13]. Data-sets were incorporated via standardized transcripts without additional technical cost to the partners. This led to a constant offline updating process of the central database with bibliographical information from the local nodes.

**Figure 1:**
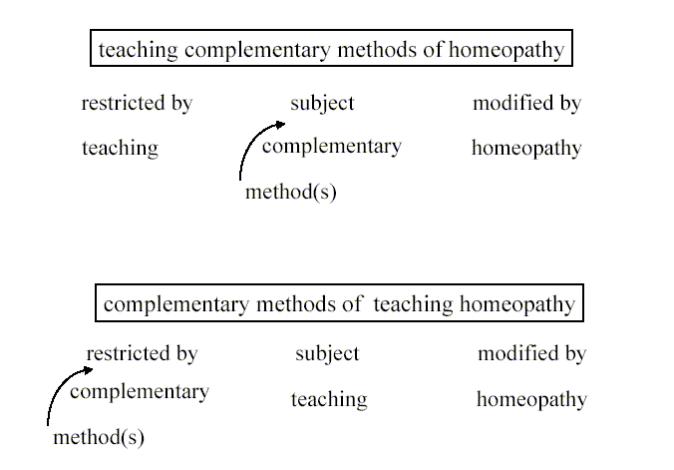
**Processing of search queries**. Example of two search queries with identical words but different meanings. By using semantical and syntactical parsing of the search phrase, the structural information of the phrase is integrated in the search algorithm, which leads to a relevance ranking of the results.

In addition to conventional search options (Author, title, keywords, publication year,...) we implemented a natural language interface with linguistic algorithms to simplify the search for users without a greater knowledge of research databases. These algorithms recognize especially the modification and restriction of a subject, explicitly formulated by the user. Even though the text consists of the same words (Fig. 1), the ranking of the search results is different [14].

**Figure 2:**
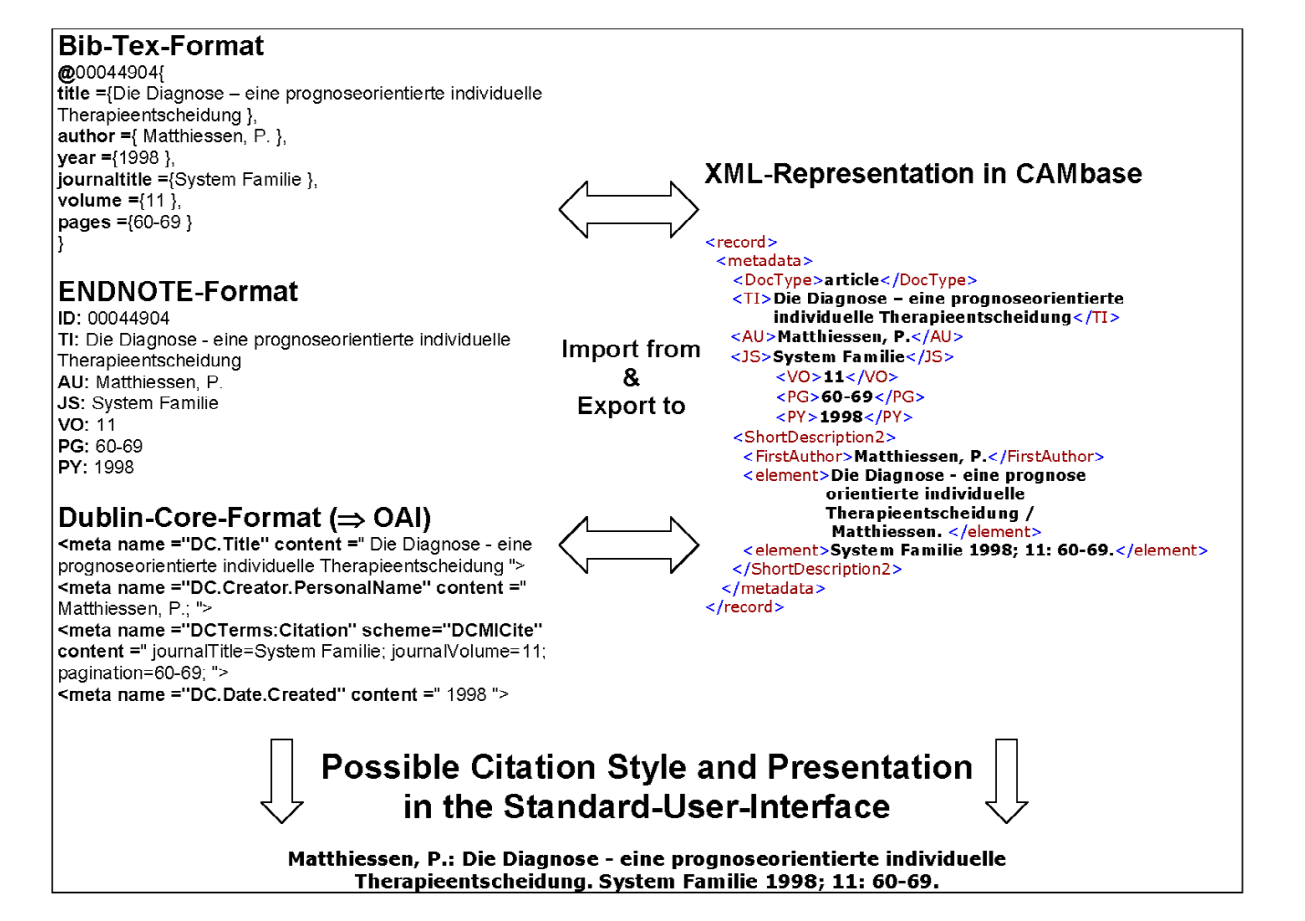
**XML-based representation of bibliographical data**. Bibliographical data of various formats (ENDNOTE, BibTeX, Dublin Core) is processed via XML-tools into a XML-representation in CAMbase, which also is the basis for processing the search queries. Output can be shown in various views like the above mentioned formats and citation styles including the short form of the Standard-User-Interface.

As bibliographical data in our case does contain several heterogenous textfragments, we decided in 2003 to use the innovative technological web-standard XML (eXtended Markup Language) [15]. With this technology we developed tools to extract structural and descriptive metadata of incoming documents and to deliver special document output styles on demand (Fig. 2). As a necessary feature we also implemented XML-interfaces for the standards given by the Open-Archives-Initiative (OAi). With this XML-based document-management CAMbase can be easily connected to national and international electronic databases and digital libraries.

For quality management we also implemented a statistical routine, which allows us to evaluate the search queries over the course of time. With this tool we are able to detect the use of different search strategies enabling us to evaluate the relevance and dynamics of CAM-related topics by means of statistical analysis, which is presented in the next section.

## Content analysis and search query statistics

At present, CAMbase covers about 80,000 bibliographical records from more than 30 journals and periodicals on complementary medicine, most of them not listed in MED LINE [16], covering CAM in general (23.0%), Anthroposophical Medicine (18.7%), Physical Therapies and Naturopathy (both 17.4%), Music Therapy (8.4%) and Homeopathy (7.4%) to mention the major fields of CAM. Some of them have a long tradition and hence play an important role for the development of Complementary Medicine particularly in Germany. We have therefore attempted to integrate not only the current but also the older issues of those journals.

**Figure 3:**
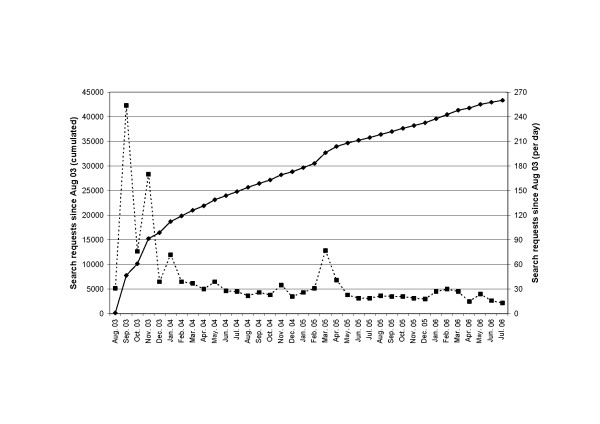
**Development of Search Queries in Cambase**. Statistics of search queries in CAMbase over a three-year period. The dotted line (right axis) gives monthly access statistics while the solid line (left axis) shows cumulative statistics in the course of time.

Since the restructuring of CAMbase to the XML-standard in August 2003, more than 43,000 search queries, an average of 38 search queries per day, were registered. Especially in the first 12 months, due to some press releases, CAMbase recorded more than 120 search queries per day, as can be seen in Fig. 3. Most of the users favoured the 'thematic search' tool especially in the first year. However, over time, other search options became more and more relevant (see Tab. 1). Especially the search for authors increased significantly from 49.0% in the first year to more than 89.0% in the second and 85.0% in the third year. In order to find out more of the areas of interest of our users, a more detailed analysis of the most frequent search terms (used more than five times) was carried out. From a total of 28,752 search terms, 3,185 (11.1%) could not be analysed. 3,522 (12.2%) search terms were about CAM-related authors. The remaining search terms (N = 22,045, 76.7%) could be classified in the following fields: 'general terms' (n = 3,147, 10.9%), 'diseases, disorders and symptoms' (n = 8,713, 30.3%), 'therapies and procedures' (n = 7,270, 25.2%), 'plants and ingredients' (n = 2,945, 10.2%).

**Table 1:** Change of search strategies of CAMbase-users

Search strategies of CAMbase users in % (N = 43,299)			
	Aug 03 – Jul 04	Aug 04 – Jul 05	Aug 05 – Jul 06

Thematic search	86.0 %	70.0 %	66.1 %
Other search strategies	14.0 %	30.0 %	33.9 %

**Figure 4:**
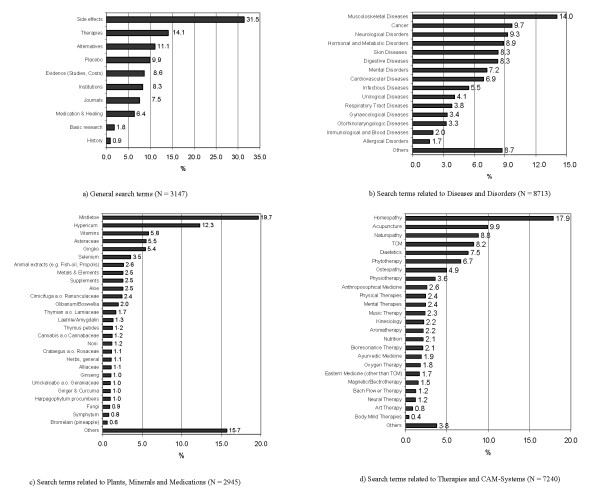
**Search topics processed in CAMbase**. Thematically grouped top-list of search topics processed in CAMbase.

The leading categories were 'homeopathy' (17.9% in the therapies group; 4.5% overall), 'muscolosceletal diseases' (14.0% in the diseases group; 4.2% overall), 'side effects' (31.4% in the general group; 3.5% overall), 'cancer' (9.7% in the diseases group; 2.9% overall) and 'acupuncture' (9.9% in the therapies group; 2.5% overall). A detailed analysis of all categories in the four fields is given in Fig. 4.

Within this three year period analysis of search terms on the one hand does reflect quite well the current trends in CAM-research (e.g. acupuncture, safety). On the other hand it also reflects the growing interest for CAM in fields such as cancer, where CAM is often requested and applied. This led us to the conclusion that apart from scientists and researchers, CAMbase is also used by physicians and patients to find out more about Complementary Therapies.

## Perspectives

Although a growing number of scientific publications on CAM can be observed in conventional databases, the complete spectrum of complementary therapies is still in need of more information about published evidence [17]. CAMbase is a first attempt to close this gap. However, there are still some unsolved problems with regards to the performance of search queries. One essential problem is the so-called *vocabulary problem *first introduced by Furnas [18]. Both the verbalization of a search query as well as the indexing of bibliographical data lacks precision, and there is little agreement between two people in classifying an object with a limited repository of terms. Particularly older literature in CAM has no or insufficient keywords or subject headings [8], and without a concise repertoire of controlled vocabulary this results in the need to build up tools which guide the user through the bibliographical landscape. Several approaches have been discussed within this field using neural-network applications like support vector machines [19], self – organizing maps [20] or the implementation of a knowledge repository [21]. We will try to connect these approaches with the linguistic algorithms already implemented in CAMbase to create a (graphical) search-tool.

Apart from the literature which has been printed, electronic versions of full text articles are becoming increasingly relevant for international research projects on CAM. Especially for physicians, who do not have access to the infrastructure of university and similar institutions, being able quickly to access not only bibliographical records on CAM but also the complete article is essential to make such a database really valid for everyday life. However, full-text archives are restricted by copyright laws which authorize storage of full text articles only if permission by authors and/or publishers has been granted. Therefore, it is necessary to build up a special library of CAM to provide articles for a full-text document-delivery service. The activities of such a library can be closely related and connected with the academic teaching of CAM at the Center of Complementary Medicine at Witten/Herdecke University. Working in close cooperation with clinicians from different fields such as Anthroposophical Medicine, Osteopathy, Naturopathy or Homeopathy, the library will not only cover specialist areas of research, but also general questions on practical health services.

**Figure 5:**
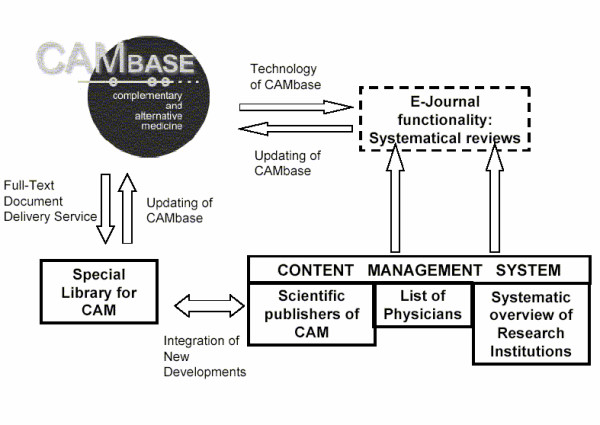
**CAM-Network**. A network of CAM around the CAMbase-architecture.

Additionally, a user might not only want to access the scientific literature of CAM, but also to look for a local research institution or a physician involved in CAM. This, in addition to bibliographical data in CAMbase, can be realized with a content management system linked to CAMbase, in which such information is stored. Hence, an extension of CAMbase towards a multi-dimensional web portal (Fig. 5) is currently under discussion.

## Authors' contributions

PFM has contributed to outlining the CAMbase project and has written parts of the introduction to the manuscript. HZ is responsible for the technical realization and has developed the semantic algorithms and contributed to the methodological parts of the manuscript. AB supervised the classification of search terms and assisted in the project by researching medical questions. CKR is documentation officer of this project and assisted in the classification of search terms. TO is project manager. He is responsible for the statistical analysis and wrote the major part of the manuscript.
